# HDAC6 regulates dental mesenchymal stem cells and osteoclast differentiation

**DOI:** 10.1186/s12903-018-0624-1

**Published:** 2018-11-21

**Authors:** Yi Wang, Zhi Yun Shi, Jin Feng, Jun Kai Cao

**Affiliations:** Department of Stomatology, Chinese PLA General Hospital, 28th Fuxing Road, Beijing, 100853 People’s Republic of China

**Keywords:** Dental mesenchymal stem cell, HDAC6, Odontogenic differentiation, Osteoclasts, Osteoclastogenesis

## Abstract

**Background:**

Dental and periodontal tissue development is a complicated process involving a finely regulated network of communication among various cell types. Understanding the mechanisms involved in regulating dental mesenchymal stem cells (MSCs) and osteoclast cell differentiation is critical. However, it is still unclear whether histone deacetylase HDAC6 is involved in dental MSCs fate determination and osteoclast differentiation.

**Methods:**

We used shRNA and siRNA knockdown to explore the role of HDAC6 in dental MSCs odontogenic differentiation and osteoclasts maturation.

**Results:**

Based on HDAC6 knockdown dental MSCs, our data suggest that HDAC6 knockdown significantly increases alkaline phosphate activity and mineralized nodules formation. Additionally, mRNA expression of odontogenic marker genes (OSX, OCN, and OPN) was induced by HDAC6 knockdown. By using HDAC6 siRNA, we knocked down HDAC6 in osteoclast precursor RAW 264.7 cells. Our data suggests that HDAC6 knockdown significantly inhibited osteoclasts differentiation. Additionally, mRNA expression of osteoclast marker genes Trap, Mmp9, and Ctsk was decreased by HDAC6 knockdown.

**Conclusions:**

Our study demonstrated that HDAC6 plays an important role in regulating dental MSCs and osteoclasts differentiation.

## Background

Bone is continuously undergoing resorption and remodeling to maintain bone volume and calcium homeostasis. Osteoblasts and osteoclasts play critical roles in regulating bone formation and resorption. Osteoblast cells are believed to derive from MSCs that originate in bone marrow [1]. MSCs are adult stem cells which can differentiate into chondrocytes, adipocytes, osteoblasts, myocytes, and hepatocytes [2, 3]. Osteoclasts are multinucleated cells differentiated from hematopoietic stem cells (HSCs) [4, 5]. Because the regulation of osteoclastogenesis and osteoblastogenesis are critical in tooth eruption and movements [6], many studies have been conducted to unveil the mechanism involved in bone cell differentiation. Broad-acting histone deacetylase (HDAC) inhibitor TAS has been reported to increase osteoblast proliferation and the transcriptional activity of Runx2 [7]. Another study demonstrated that HDAC inhibitor FR901228 decreased the maturation of osteoclasts [8]. However, there is still much to be understood about the specific role of individual HDACs in the regulation of dental bone resorption and regeneration.

Studies have shown that dental MSCs have multipotency for odontogenic, osteogenic, chondrogenic, and adipogenic potential under different culture conditions [9–11]. Therefore, dental MSCs are a promising treatment approach for clinical applications in dental regeneration and craniofacial therapies. The features of dental MSCs are influenced by various factors including cytokines, cell passages, and oxygen concentration. In the last decade, our knowledge about molecular contributors to MSCs differentiation has also increased. Bone lineage commitment is reportedly regulated by the expression of transcription factor RUNX2, which further promotes expression of alkaline phosphatase (ALP), osterix (OSX), osteopontin (OPN), and osteocalcin (OCN) [12]. In addition, several transcription factors and signal pathways have been demonstrated to regulate MSCs differentiation [13, 14].

Osteoclasts are multinucleated cells differentiated from monocytes or macrophages that absorb bone matrix. Osteoclast differentiation is regulated by the tumor necrosis factor (TNF) family cytokine, receptor activator of nuclear factor (NF)-κB ligand (RANKL), and macrophage colony-stimulating factor (M-CSF) [15–17]. Studies have suggested that TNF receptor-associated factor 6 (TRAF-6), NF-κB and c-fos are also essential molecules for osteoclast genesis [17]. In addition, genome-wide screening has provided insights into additional genes that are involved in the regulation of osteoclast differentiation.

Histone deacetylases (HDACs) are a group of conserved enzymes that remove acetyl groups from lysine side chains of both histones and nonhistone proteins. HDACs are reportedly involved in the regulation of bone formation and maintenance [18, 19]. HDAC6, a unique protein that belongs to class IIb of HDACs, can shuttle between the cytoplasm and nucleus. Previous studies have demonstrated that HDAC6 inhibitors accelerate osteoblast maturation and suppress osteoclast differentiation [20]. HDAC6-deficient mice develop a slightly enlarged tibia and increased bone mineral density [21]. The Rho–mDia2–HDAC6 pathway is reportedly involved with osteoclast maturation by controlling podosome patterning through microtubule acetylation in osteoclasts [22]. While these studies indicate a role for HDAC6 in bone growth and resorption, the role of HDAC6 in dental MSCs and osteoclast differentiation is still unclear. In this study, we demonstrate that HDAC6 knockdown in dental MSCs can promote osteoblast maturation. We further demonstrate that HDAC6 loss can inhibit osteoclast differentiation. Thus, our findings suggest that HDAC6 plays an important role in dental bone growth and maintenance.

## Methods

### Human DPSCs isolation and culture

All patients provided informed consent according to the guidelines of the Ethics Committee of Chinese PLA General Hospital. Dental pulp was obtained from normal third molars of three adults donors (19–29 years of age) at the Dental Clinic of Chinese PLA General Hospital. Human DPSCs were isolated as previously reported [23]. Cells were cultured in a humidified 5% CO_2_ incubator at 37 °C in alpha-modified Eagle’s medium (Invitrogen) supplemented with 15% fetal bovine serum (Invitrogen), Gibco MEM non-essential amino acids (Invitrogen), 2 mmol/L L-glutamate, 100 units/mL penicillin, and 100 units/mL streptomycin. The RAW 264.7 cell line was cultured in a humidified 5% CO_2_ incubator at 37 °C in RPMI-1640 medium (Invitrogen) supplemented with 5% fetal bovine serum, 100 units/ml penicillin, and 100 units/ml streptomycin.

### Viral infection

Lentiviral expression vector was constructed using pLKO.1 vector (Addgene). The sequence for scramble (control) was 5’-CCTAAGGTTAAGTCGCCCTCG-3′. The target sequences for shRNA were *HDAC6*sh1, 5’-CATCCCATCCTGAATATCCTT-3′ and *HDAC6*sh2, 5’-GCACAGTCTTATGGATGGCTA-3′. The insert was subcloned into pLKO.1 using Agel and EcoRI sites. Lentivirus production was performed according to the protocol provided by Addgene. Forty-eight hours after the transfection, the media containing viruses were collected and concentrated by ultracentrifugation.

Dental MSCs were seeded overnight and then infected with lentivirus in the presence of polyybrene (6 μg/mL; Sigma-Aldrich) for 24 h. The cells were then selected with puromycin for 72 h. Puromycin-resistant clones were cultured, and HDAC6 expression was detected by real-time RT-PCR. In rescue experiments, HDAC6-knockdown dental MSCs were infected with adenovirus (Sigma-Aldrich, St. Louis, MO, USA) containing flag-tagged HDAC6.

### siRNA transfection

siRNAs were synthesized by Invitrogen and transfected with a Lipofectamine 2000 reagent (Invitrogen) at a final concentration of 40 nM. The target sequences for siRNA were *HDAC6*siRNA1: 5’-GCACCAUGGUCAAGGAACA-3′ and *HDAC6*siRNA2: 5’-CCAAUCUAGCGGAGGUAAA -3′.

### ALP staining and ALP activity assay

Dental MSCs were cultured in a osteogenic medium containing 100 μmol/L ascorbic acid, 2 mmol/L b-glycerophosphate, and 10 nmol/L dexamethasone. After induction, cells were washed with PBS and fixed with 4% paraformaldehyde and incubated in BCIP/NBT solution (Beyotime, Shanghai, China) in the dark. Areas that stained purple were regarded as positive. ALP activities were determined using p-nitropheyl phosphate (Sigma) as described previously [24].

### Alizarin red staining (ARS)

Dental MSCs were cultured in osteogenic medium. After 3 weeks, cells were washed with PBS, and fixed with 70% ethanol at 4 for 1 h. Thereafter, the cells were stained with 2% Alizarin Red (Sigma-Aldrich). To quantify the calcium mineral deposition, Alizarin Red was destained with 10% cetylpyridinium chloride in 10 mmol/L sodium phosphate for 30 min at room temperature. Alizarin Red concentration was determined by absorbance at 562 nm on a microplate reader using a standard calcium curve in the same solution. The final calcium level in each group was normalized with the total protein concentration obtained from a duplicate plate.

### Osteoclast induction and trap staining

RAW 264.7 Cells were seeded in a 12-well culture plate (Corning) with OC differentiation medium containing 100 ng/mL recombinant RANKL (PeproTech). After 6 days, the medium was removed and the cells were washed with PBS. The cells were then subjected to TRAP staining (Sigma-Aldrich) following the manufacturer’s instructions to confirm their OC identity.

### Real-time RT-PCR

Total RNA was extracted using TRIzol reagents (Invitrogen) and was transcribed with PrimeScript RT reagent kit (Takara Bio, Inc., Kusatsu, Japan). cDNA amplification and detection were performed using the Bio-Rad iQ5 real-time PCR system (Bio-Rad, Hercules, CA, USA) using SYBR Premix Ex Taq kit (Takara) and specific primers. The primers sequences are listed below:

- *18S* rRNA-forward: 5’-CGGCTACCACATCCAAGGAA-3′;
- *18S* rRNA-reverse, 5’-GCTGGAATTACCGCGGCT-3′.
- *HDAC6*-forward: 5’-TCAGGTCTACTGTGGTCGTT-3′;
- *HDAC6*-reverse: 5’-TCTTCACATCTAGGAGAGCC-3′.
- *OSX*-forward: 5’-CGCTTTGTGCCTTTGAAA-3′;
- *OSX*-reverse: 5’-CCGTCAACGACGTTATGC-3′.
- *OCN*-forward: 5’-CAGACACCATGAGGACCATC-3′;
- *OCN*-reverse: 5’-GGACTGAGGCTCTGTGAGT-3′.
- *OPN*-forward: 5’-ATGATGGCCGAGGTGATAGT-3′;
- *OPN*-reverse: ACCATTCAACTCCTCGCTTT-3′.
- *ALP*-forward: 5’-GACCTCCTCGGAAGACACTC-3′;
- *ALP*-reverse: 5’-TGAAGGGCTTCTTGTCTGTG-3′.
- Mouse *β-actin*-forward: GATGCCAGCGACAAGAGGTT-3′;
- Mouse *β-actin*-reverse: 5’-CATACCAGGGGATGTTGCGAA-3′.
- Mouse *HDAC6*-forward: 5’-GAAGGAGGAGCTGATGTTGG-3′;
- Mouse *HDAC6*-reverse: 5’-TCATGTACTGGGTTGTCTCCAT-3′.
- Mouse *TRAP*-forward: 5’-GAAGAAGACTCACCAGAAGCAG-3′;
- Mouse *TRAP*-reverse: 5’-TCCAGGTTATGGGCAGAGATT-3′.
- Mouse *Mmp9*-forward: 5’-CAGGAGAGGCATTATGAGCA-3′;
- Mouse *Mmp9*-reverse: 5’-GGTACTTTCCTGGTTCGCAT-3′.
- Mouse *Ctsk*-forward: 5’-CTGGACAGCCAGACACTAAAG-3′;
- Mouse *Ctsk*-reverse: 5’-CTCGCGGCAAGTCTTCAGAG-3′.

### Western blot

Cells were solubilized in ice-cold RIPA buffer (Invitrogen) supplemented with complete protease inhibitor tablets (Roche, Basel, Switzerland). 40 μg of total protein was resolved by SDS-PAGE and transferred to PVDF membranes (Millipore, Burlington, MA, USA). Membranes were blotted with antibodies against HDAC6 (Abcam, Cambridge, UK) and α-tubulin (Cell Signaling Technology, Danvers, MA, USA) and HRP-conjugated secondary antibodies. The protein bands were visualized by the ECL system according to the manufacturer’s instructions and exposed to x-ray film.

### Statistics

Data are presented as the mean ± SD of at least three independent experiments. Differences between groups were evaluated using Student’s *t*-test. *p*-value < 0.05 was considered significant.

## Results

HDAC6 inhibits odontogenic differentiation of dental MSCs.

**Fig. 1:**
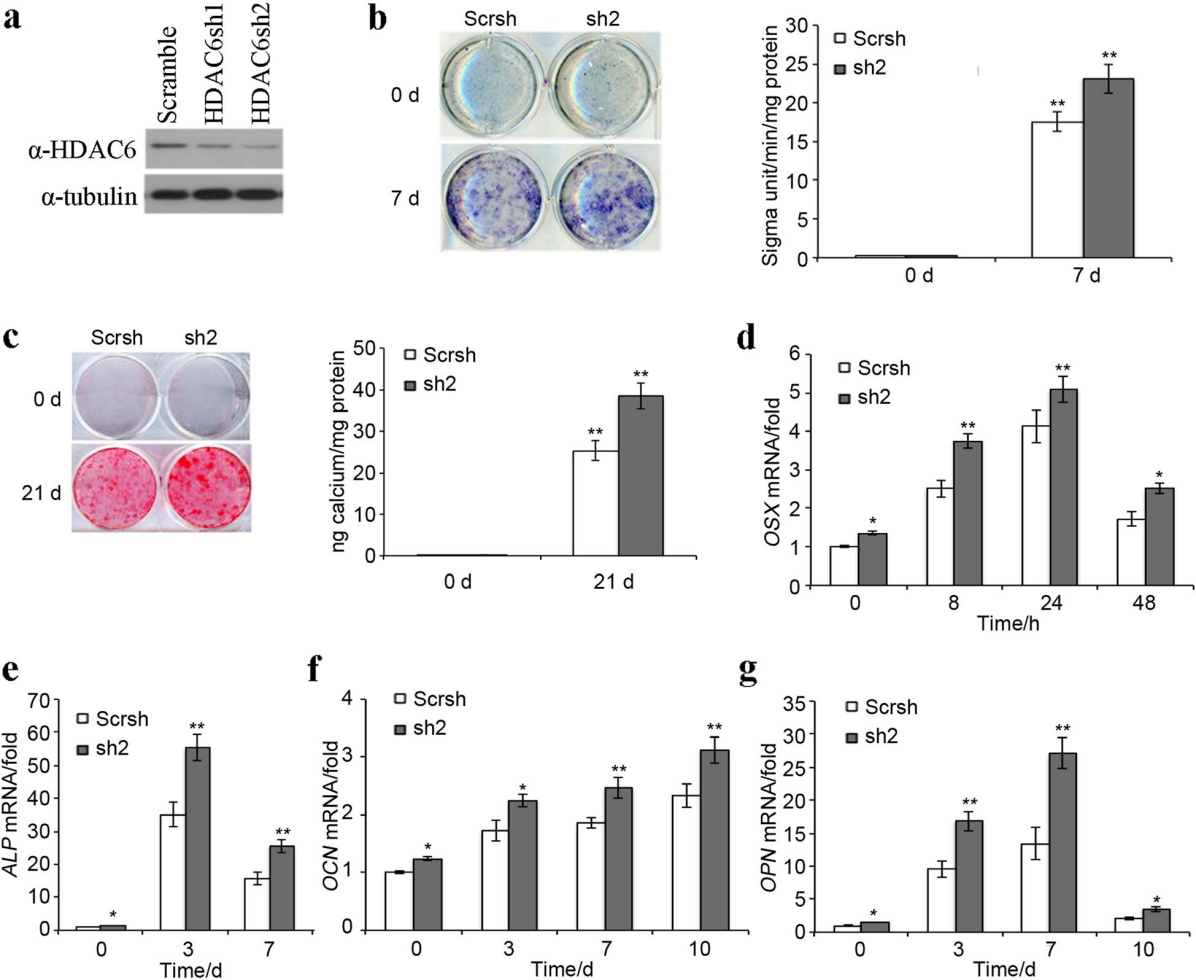
HDAC6 knockdown increases odontogenic potential of dental MSCs. **a** ShRNA knockdown efficiency of HDAC6 was confirmed by Western blot. **b** Knockdown of HDAC6 increased ALP activity in dental MSCs. **c** Knockdown of HDAC6 promoted mineralization in MSCs. **d** Knockdown of HDAC6 promoted *OSX* expression in dental MSCs. **e** Knockdown of HDAC6 promoted *ALP* expression in dental MSCs. **f** Knockdown of HDAC6 promoted *OCN* expression in dental MSCs. **g** Knockdown of HDAC6 promoted *OPN* expression in dental MSCs

Previous studies have suggested that the inhibition of HDAC6 could promote BMSCs differentiation. Dental MSCs are reportedly capable of differentiating into odontoblasts similar to BMSCs. Therefore, we designed experiments to explore the function of HDAC6 in dental MSCs differentiation. Dental MSCs were infected with lentivirus-carrying *HDAC6* shRNAs to generate HDAC6 knockdown cell lines. The knockdown efficiency of two different *HDAC6* shRNAs was confirmed by Western blot (Fig. 1a). As *HDAC6* sh2 showed higher knockdown efficiency, we chose dental MSCs infected with HDAC sh2 for use in further experiments. When these cells were induced with bone formation medium, MSC/sh2 cells showed increased bone formation as demonstrated by ALP staining and activity on day 7 (Fig. 1b). Furthermore, HDAC6 knockdown also increased the formation of mineralized nodules after prolonged induction with bone formation medium for 21 days as demonstrated by ARS staining (Fig. 1c). By real-time RT-PCR, we examined mRNA expression of odontogenic marker genes at different time points after bone formation induction. Our results showed that HDAC6 knockdown significantly induced the expression of *OSX* (Fig. 1e), *ALP* (Fig. 1f), *OCN* (Fig. 1g), and *OPN* (Fig. 1h). Taken together, these results indicate that HDAC6 plays an important role in odontogenic differentiation of MSCs.

Rescue of HDAC6 inhibits odontogenic differentiation of dental MSCs.

**Fig. 2:**
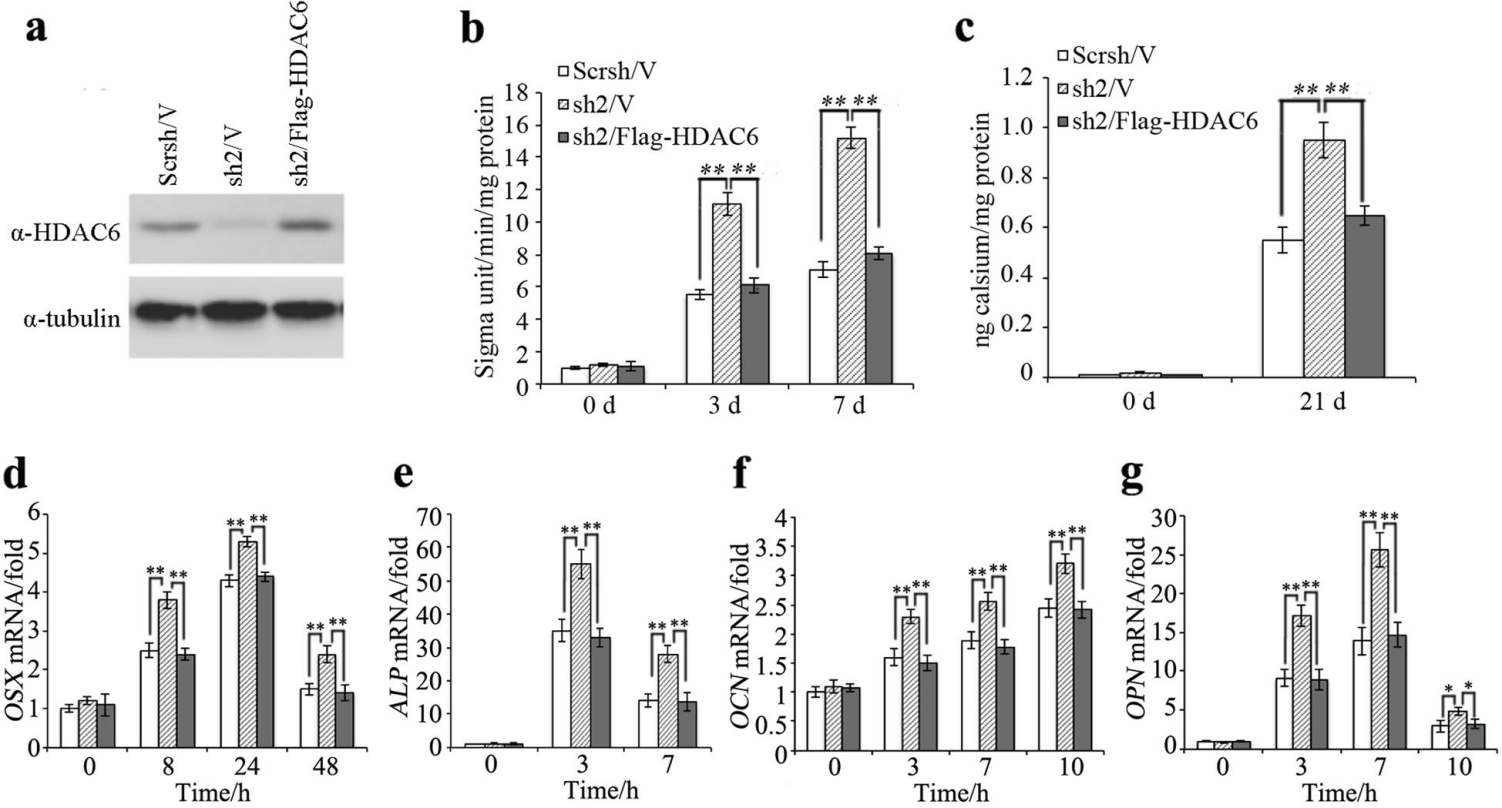
The rescue of HDAC6 inhibits odontogenic potential of dental MSCs. **a** Overexpression of HDAC6 in MSCs that express HDAC6 shRNA is confirmed by Western blot. **b** Overexpression of Flag-HDAC6 inhibited ALP activities in MSC differentiation. **c** Overexpression of Flag-HDAC6 inhibited mineralization of MSC differentiation. **d** Overexpression of Flag-HDAC6 decreased *OSX* expression in MSC differentiation. **e** Overexpression of Flag-HDAC6 decreased *ALP* expression in MSC differentiation. **f** Overexpression of Flag-HDAC6 decreased *OCN* expression in MSC differentiation. **g** Overexpression of Flag-HDAC6 decreased *OPN* expression in MSC differentiation

To validate our finding from HDAC6 knockdown dental MSCs, we restored HDAC6 expression in MSCs expressing *HDAC6* shRNA by infecting the cells with lentivirus-expressing Flag-HDAC6. Expression of HDAC6 was confirmed by Western blot (Fig. 2a). When *HDAC6* sh2/Flag-HDAC6 cells were cultured with osteogenic medium, the cells showed similar bone formation capacity to control cells (scrsh/V), whereas *HDAC6* sh2/V cells showed impaired bone formation capacity in ALP (Fig. 2b) and ARS assays (Fig. 2c). Similarly, the expression of *OSX*, *ALP*, *OCN*, and *OPN* as a result of HDAC6 knockdown was impaired after HDAC6 expression was restored (Fig. 2d–g), indicating that HDAC6 inhibits odontogenic differentiation.

### Knockdown of HDAC6 inhibits osteoclast cells differentiation

**Fig. 3:**
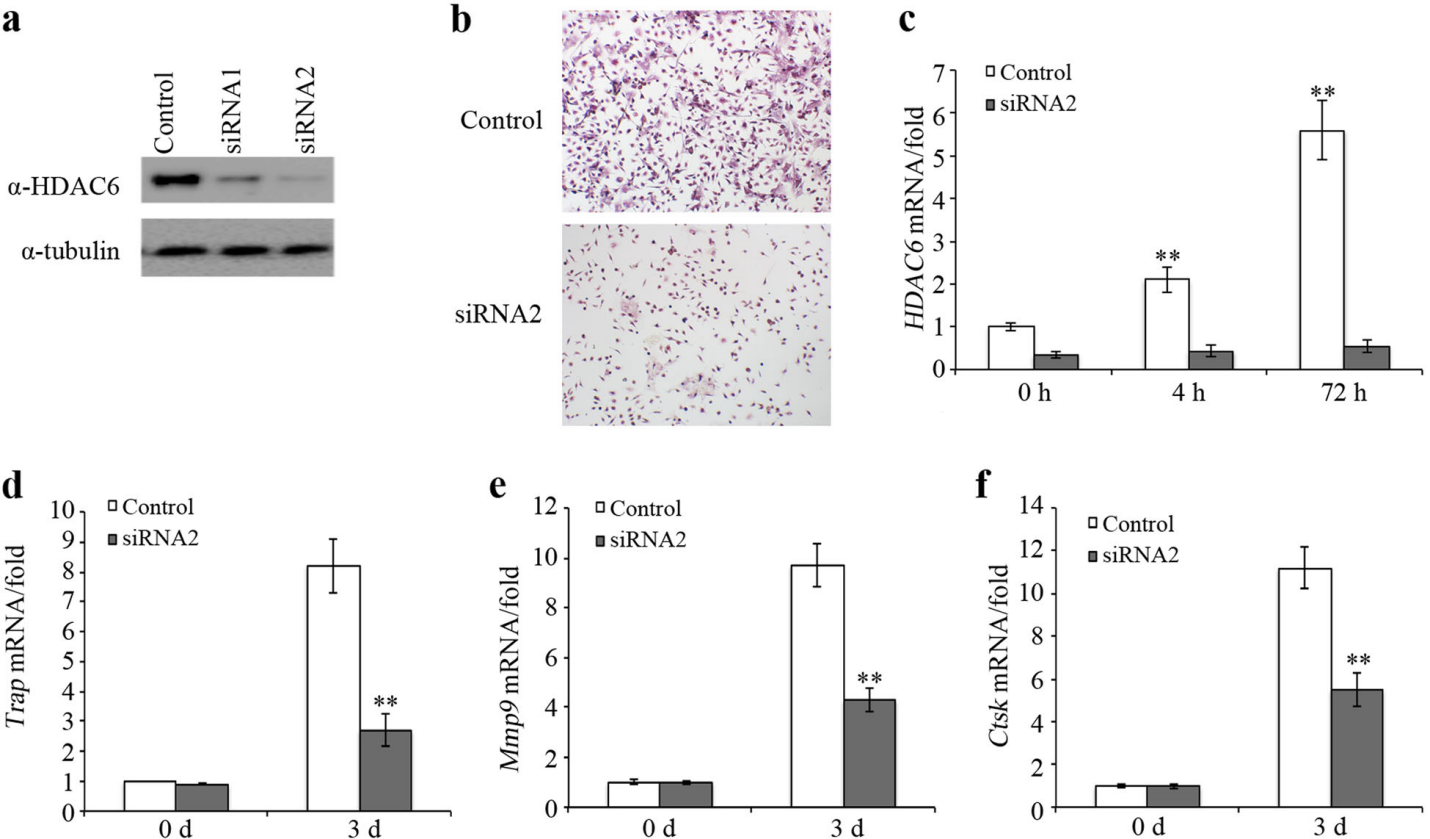
HDAC6 knockdown inhibited osteoclasts differentiation. **a** siRNA knockdown of HDAC6 is confirmed by Western blot. **b** Knockdown of HDAC6 inhibited osteoclast differentiation. **c** Expression of HDAC6 was induced by RANKL in RAW 264.7 cells. **d** Knockdown of HDAC6 decreased *Trap* expression. **e** Knockdown of HDAC6 decreased *Mmp9* expression. **f** Knockdown of HDAC6 decreased *Ctsk* expression

HDAC6 inhibitors inhibit osteoclast cell differentiation. Therefore, we designed an experiment to study the role of HDAC6 in osteoclast differentiation. Macrophage line were transfected with control siRNA and *HDAC6* siRNAs. The knockdown efficiency of *HDAC6* was detected by Western blot (Fig. 3a). We chose *HDAC6* siRNA2 with higher knockdown efficiency for further experiments. After transfection with *HDAC6* siRNAs, RAW 264.7 cells were induced with RANKL to activate differentiation. Compared with the control line, RAW 264.7 cells transfected with *HDAC6* siRNA showed a lower capability to form mature osteoclasts (Fig. 3b). Interestingly, the expression of *HDAC6* was also induced by RANKL in RAW 264.7 cells transfected with control siRNA (Fig. 3c). *Trap*, *Mmp9*, and *Ctsk* genes play critical roles in osteoclast differentiation [25–27] Furthermore, we found that RAW 264.7 cells transfected with *HDAC6* siRNA2 showed lower expression levels of *Trap* (Fig. 3d), *Mmp9* (Fig. 3e), and *Ctsk* (Fig. 3f).

### Rescue of HDAC6 restores osteoclast differentiation

**Fig. 4:**
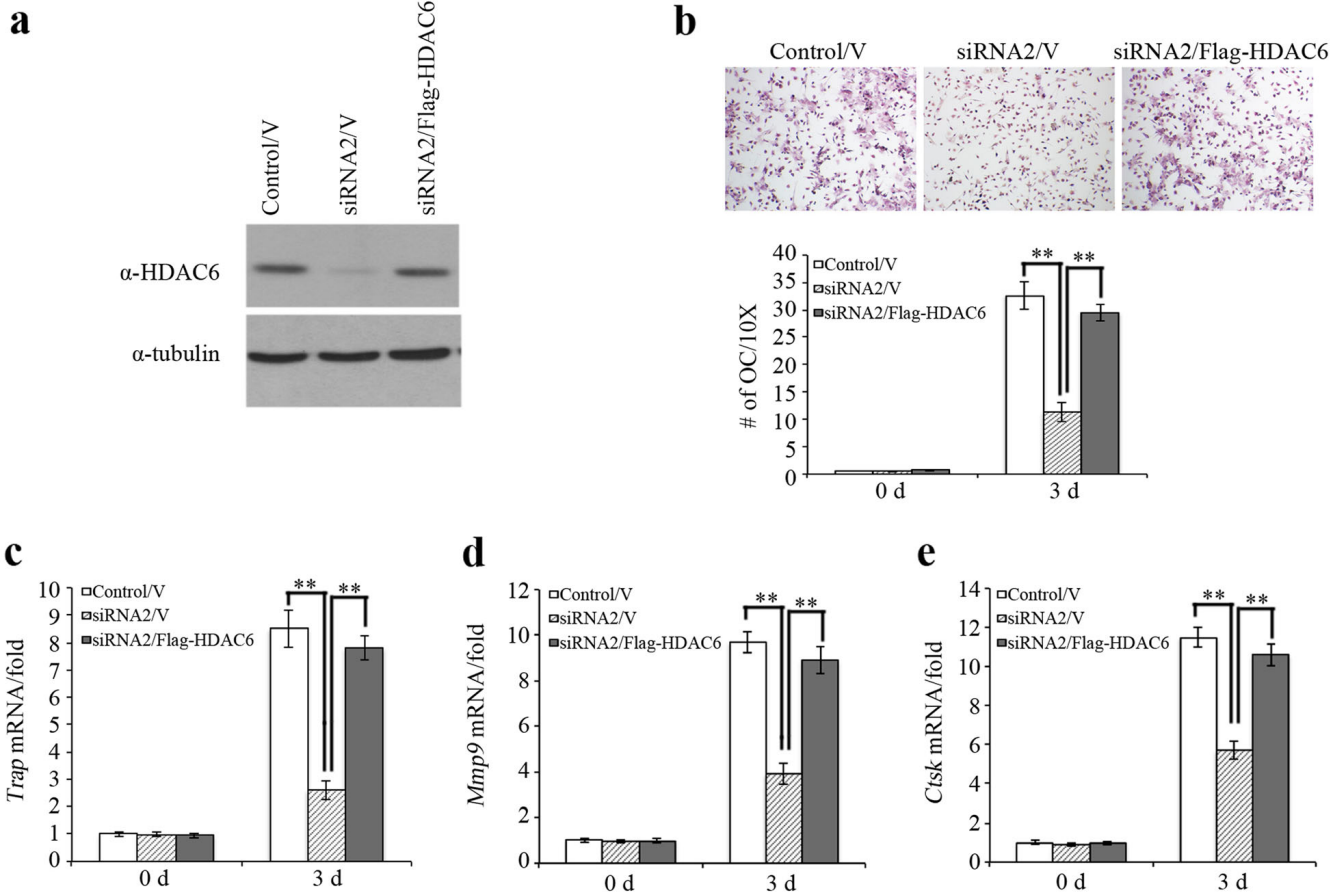
Rescue of HDAC6 restored osteoclast differentiation. **a** Overexpression of HDAC6 in osteoclasts that transfected with HDAC6 siRNA. **b** Overexpression of HDAC6 restored osteoclast differentiation. **c** Overexpression of HDAC6 increased *Trap* expression. **d** Overexpression of HDAC6 increased *Mmp9* expression. **e** Overexpression of HDAC6 increased *Ctsk* expression

We restored HDAC6 expression in RAW 264.7 cells transfected with *HDAC6* siRNA by infecting the cells with adenoviruses expressing Flag-HDAC6. HDAC6 expression was confirmed by Western blot (Fig. 4a). When HDAC6 siRNA/Flag-HDAC6 cells were induced with RANKL, the cells showed similar osteoclast differentiation capacity to the control cells (control siRNA/V) in a trap staining assay (Fig. 4b). Similarly, the decreased expression of *Trap*, *Mmp9*, and *Ctsk* as a result of HDAC6 knockdown was rescued after HDAC6 expression was restored (Fig. 4c–e), indicating that HDAC6 promotes osteoclast differentiation.

## Discussion

Using dental MSCs to regenerate forms and functions of teeth is one of the important goals in dental tissue engineering, although additional fields of application are quickly emerging. Previous studies have discovered mechanisms involved in regulating odontogenic differentiation potential in dental MSCs [28, 29]. While dental MSCs are similar to BMSCs in their potential to differentiate into mineralized tissues, dental MSCs have more restricted differentiation than bone marrow cells have in vivo. It has been reported that dental MSCs are more committed to odontogenic than to osteogenic development in vitro [30]. Stem cells derived from dental tissue have been used for tissue engineering studies to assess their potential in pre-clinical application [31–34]. Therefore, a better understanding of the molecular mechanisms underlying cell differentiation of dental MSCs is of significant interest in regenerative medicine.

Osteoclasts are highly specialized migratory cells that regulate bone resorption. They are critical for normal skeletal growth and the maintenance of bone integrity throughout life. Overactive osteoclasts may cause several bone diseases including Paget’s disease in bone, juvenile Paget’s disease, expansile skeletal hyperphosphatasia, and familial expansile osteolysis [35–39]. Dental problems resulting from osteoclast activation have long been recognized. To improve our understanding and treatment of dental abnormalities and osteoclast diseases, it is critical to study mechanisms involved in regulation of osteoclasts differentiation.

HDACs can induce specific changes in gene expression by deacetylating both histone and non-histone proteins. HDAC inhibitors have been used in clinics for treatment of cancer, epilepsy, and bipolar disorder. In 1993, Iwami and Moriyama demonstrated that the HDAC inhibitor NaB could induce ALP expression in a MC3T3-E1 pre-osteoblast cell line [40]. Several studies suggest the HDAC inhibitor TSA has stimulatory effects on osteoblast cell lines, primary calvarial osteoblasts, and in calvarial organ cultures [7, 41]. TSA and NaB inhibit RANKL-mediated osteoclast differentiation from hematopoietic precursors [42]. Furthermore, several HDACs play important roles in bone development and physiology; HDAC1, HDAC3, HDAC6, and HDAC7 are involved with osteoblastogenesis through cooperation with, or inhibition of, Runx2 [43–46]. There is still much to learn about the roles of HDACs in osteoclast differentiation. Interestingly, a study from 2011 showed that HDAC3 and HDAC7 play opposite roles in osteoclast differentiation [47]. Suppression of HDAC7 facilitated osteoclastogenesis and increased osteoclast size [48]. HDAC9 knockout mice had elevated osteoclast numbers and bone resorption indices [49]. Class III HDAC Sirt1 also inhibited osteoclast differentiation in a conditional deletion Sirt1 mouse model [50].

As a member of the class II HDAC family, HDAC6 predominantly localizes in the cytoplasm. It plays an important role in regulating cell motility by deacetylating a-tubulin and cortactin [51, 52]. HDAC6 also modulates Hsp90-dependent activation of the glucocorticoid receptor through deacetylating Hsp90 [53, 54]. Loss of HDAC6 increased the formation of ossified bone in TDII embryos, but it did not lead to significantly different bone mineral densities at later ages [55, 56]. Specific inhibition of HDAC6 with Tubastatin or AC1215 increased osteoblast formation and osteoclast inhibition [20]. Taking into account previous published results, we have shown that HDAC6 knockdown promotes dental MSCs differentiation by using shRNA. By employing a small interfering RNA technique, we also knocked down the expression of HDAC6 in murine osteoclast precursor cells line RAW 264.7. The knockdown of HDAC6 in RAW 264.7 cells inhibited osteoclasts formation under RANKL stimulation. However, it is still not clear whether HDAC6 could regulate osteoclast precursor proliferation. Although further studies are required to understand the detailed molecular mechanisms by which HDAC6 regulates the expression of osteoclast marker genes, our results shed light on the role of HDAC6 in dental MSCs and osteoclasts differentiation. Together with previous findings, our study suggests that HDAC6 is a potential regulator for bone growth and maintenance.

## Conclusions

We expect that HDAC6 knockdown will lead to inhibition of osteoclastogenesis and the promotion of dental MSCs odontogenic differentiation. Our findings give new insight into the role of HDACs in tooth development and disease and suggest HDAC6 as a novel therapeutic target for disease treatment and drug development. HDAC6 knockout mice appear to be healthy, and highly specific HDAC inhibitors appear to be well tolerated in clinical trials. The inhibition of HDAC6 may offer health advantages under some dental conditions. In future studies, it will be important to determine how HDAC6 is involved with networks regulating dental cell differentiation.

## Abbreviations

ALP: Alkaline phosphatase
ARS: Alizarin red staining
HDACs: Histone deacetylases
MSCs: Mesenchymal stem cells
OCN: Osteocalcin
OPN: Osteopontin
OSX: Osterix
RANKL: Receptor activator of nuclear factor (NF)-κB Ligand
TNF: Tumor necrosis factor
TRAF-6: Receptor-associated factor 6
